# Transcriptomic and Metabolomic Approaches Deepen Our Knowledge of Plant–Endophyte Interactions

**DOI:** 10.3389/fpls.2021.700200

**Published:** 2022-01-27

**Authors:** Xue-liang Chen, Mei-chen Sun, Sun-li Chong, Jin-ping Si, Ling-shang Wu

**Affiliations:** State Key Laboratory of Subtropical Silviculture, Zhejiang A&F University, Hangzhou, China

**Keywords:** metabolome, plant–endophyte interaction, plant growth promotion (PGP), stress resistance, transcriptome

## Abstract

In natural systems, plant–symbiont–pathogen interactions play important roles in mitigating abiotic and biotic stresses in plants. Symbionts have their own special recognition ways, but they may share some similar characteristics with pathogens based on studies of model microbes and plants. Multi-omics technologies could be applied to study plant–microbe interactions, especially plant–endophyte interactions. Endophytes are naturally occurring microbes that inhabit plants, but do not cause apparent symptoms in them, and arise as an advantageous source of novel metabolites, agriculturally important promoters, and stress resisters in their host plants. Although biochemical, physiological, and molecular investigations have demonstrated that endophytes confer benefits to their hosts, especially in terms of promoting plant growth, increasing metabolic capabilities, and enhancing stress resistance, plant–endophyte interactions consist of complex mechanisms between the two symbionts. Further knowledge of these mechanisms may be gained by adopting a multi-omics approach. The involved interaction, which can range from colonization to protection against adverse conditions, has been investigated by transcriptomics and metabolomics. This review aims to provide effective means and ways of applying multi-omics studies to solve the current problems in the characterization of plant–microbe interactions, involving recognition and colonization. The obtained results should be useful for identifying the key determinants in such interactions and would also provide a timely theoretical and material basis for the study of interaction mechanisms and their applications.

## Introduction

Plant–microbe interactions have occurred throughout the evolutionary history of the plants. The fossil record provides evidence that, over 450 million years since plants were first established on land, almost all plants in the natural ecosystem have been colonized by one or more microbial symbionts (Redecker et al., 2000; Krings et al., 2007; Bonfante and Genre, 2010; Kawaguchi and Minamisawa, 2010; Atsatt and Whiteside, 2014; Genre et al., 2020). Over this lengthy span of evolution, symbiotic microbes might have played a major role within the hosts to overcome the environmental changes. The symbiotic fungi or bacteria that live asymptomatically within a healthy plant tissue are called endophytes, and normally, these can provide benefits to their host in contrast to parasites (Khare et al., 2018; Sabra et al., 2018; Afzal et al., 2019; Mishra et al., 2021), by promoting the growth of host plants (Johnson et al., 2003; Rodriguez et al., 2008), eliciting metabolite production (Strobel et al., 2004; Kaul et al., 2012; Kusari et al., 2014; Wang et al., 2015), and mediating stress relief in plants (Yan et al., 2019). Thus, plant–endophyte interactions greatly contribute to the improved plant fitness. However, the interaction mechanism is complicated, and it remains difficult to clarify at the biochemical, physiological, and molecular levels why and how endophytes can invade *via* the defense systems of host plants and co-exist with them without causing any illness (Faeth and Fagan, 2002).

Early research on plant–endophyte interactions relied on host agronomic characteristics, composition determination, and strains’ isolation, cultivation, and characterization when speculating about the interaction mechanism involved. Nowadays, with the rapid development in high-throughput sequencing, the omics technologies (at the genome, transcriptome, and proteome levels) can provide new insights to elucidate the mechanism of interaction in a given system (Xu et al., 2021). Moreover, when coupled to metabolomic studies, omics-type investigations can gradually resolve aspects of the relationships between endophyte infection, metabolite accumulation, and stress mitigation in host plants. Transcriptomics uses next-generation sequencing (NGS) technology to reveal the presence and quantity of the individual RNA molecules in biological samples (Wang et al., 2009; Li and Li, 2018). Metabolomics is used to identify and quantify the changes in metabolites due to the deletion or overexpression of a given gene (Oliver et al., 1998; Wishart, 2007; Chen F. et al., 2019), including both endogenous metabolites that are naturally produced by an organism and by the exogenous chemicals that are not (Nordström et al., 2006). The combination of transcriptome and metabolome results can help to reveal the relationship between plants and their endophytes. This review aims to outline the use of omics techniques as an effective means to characterize the plant–endophyte interaction mechanism, and to identify the key factors that determine the effectiveness of this interaction. This work will provide a theoretical basis for elucidating the interaction mechanism, and the findings should prove useful for future applications, such as in exploring the relationships between other unknown plants and microbial species.

## Roles of Plant–Endophyte Interactions

Since “endophyte” was first defined in 1809, endophytic microbes, including parasitic, mutualistic, and latent pathogenic fungi and bacteria have been continuously discovered. Mounting evidence points to the critical roles that endophytes played in the host plants. Thus, plant–endophyte interactions have become a research hotspot. Many studies have demonstrated that the relationships of endophytes living in plants ranges from mutualistic, where both plant and microbe benefit, to parasitic, where the microbe receives some benefit from the interaction at the expense of the host. Both mutualistic and parasitic interactions can be considered as symbiotic, according to the original definition of symbiosis. Latent pathogens might show pathogenicity only at certain stages of their life cycle or under specific circumstances, and the extent of damage caused by pathogens can greatly vary depending on the length of time they spend in their pathogenic phases (e.g., causing necrotic lesions) or in asymptomatic parasitic or mutualistic phases. Accordingly, microbes can transition between the trophic states of pathogenesis and mutualism and/or between mutualism and parasitism in response to internal host signals or environmental factors (Redman et al., 2001; Drew et al., 2021). Further, many convergent strategies are supported by symbiotic signaling and infection modules (Delaux and Schornack, 2021) between different endophytes and hosts. Here, we attempt to clarify the roles of symbiosis and pathogenesis as gleaned from the past and more recent studies.

### Symbiosis

The term “symbiosis” simply means reciprocal interactions between plants and microbiota in narrow sense (Delaux and Schornack, 2021). In this context, the microbe enables the host plant to absorb nutrients (Oldroyd and Leyser, 2020) and mediates its stress tolerance (Carrión et al., 2019; Trivedi et al., 2020), thereby improving plant growth (Vandana et al., 2021). In return, the host plant actively recruits microbes to accelerate their colonization (Batstone et al., 2020) and protects them against autoimmunity (Huang et al., 2021) until the next generation. The symbiosis of plant and microbes entails several typical interaction models, including those of extracellular (ectomycorrhizas), intercellular (associations with cyanobacteria), and intracellular symbiosis [arbuscular mycorrhizas (AM), orchid mycorrhizas (OM), ericoid mycorrhizas (ERM), and nitrogen-fixing symbioses of rhizobia, and *Frankia* strains]. In AM and root nodule symbioses, an intracellular symbiotic interaction is established based on a common symbiosis signaling pathway (CSSP) governed by three core genes, namely *SymRK*, *CCaMK*, and *CYCLOPS* (Genre et al., 2020; Radhakrishnan et al., 2020). A recent study by Genre et al. (2020) tentatively links the CSSP to intracellular symbioses between plants and bacteria and fungi during the evolution of plants. Hence, intracellular symbiosis relationship is presumed to be most conserved at the evolutionary level, making it worthy of an in-depth study.

Two legumes forming nitrogen-fixing root nodules (namely, *Lotus japonicus* and *Medicago truncatula*) have served as the typical model plants to study the intracellular symbiotic interaction (Roy et al., 2020). The rhizobia enabled the nodulation to allow plants’ utilization of air dinitrogen under resource-poor conditions (Cook et al., 1997). The progress of nodulation involves several different incorporated lifestyles, including rhizosphere growth, root colonization, bacterial infection, N_2_-fixing bacteroids, and release from most types of legume nodules (Wheatley et al., 2020). Throughout the phase of growth in the rhizosphere, the rhizobia secret exopolysaccharide (EPS), amino acid, and cold shock protein compounds to respectively promote their entanglement with root hairs, transport, and survivability. For the uptake into plant roots and then into nodule primordium cells, followed by the differentiation into nitrogen fixing bacteroids, rhizobia depend on highly decorated lipochitooligosaccharide (LCO) Nod factors recognized by the extracellular lysine motif (LysM) domains of plant receptors and receptor kinases, homologs of the chitin elicitor receptor kinase 1 (CERK1) of *Arabidopsis* (Bozsoki et al., 2020). In response to the interaction with rhizobia, the roots of leguminous plants secrete a variety of amino acids and key compounds for the synthesis of purine, S-adenosyl-methionine, heme, vitamin B12, CoA, and riboflavin, all of which might be used by rhizobia. In particular, el Zahar Haichar et al. (2014) and Wheatley et al. (2020) identified a 5-aminoimidazole-4-carboxamide-1-β-D-ribofuranosyl 5′-monophosphate in the roots of legumes, a substance able to supplement the rhizobial purine biosynthesis mutants during growth in the rhizosphere. Further, nodulation is controlled by the SHR-SCR module for inducing cortical cell division that is regulated by the nodule inception (NIN) transcription factor, the transcription of which is activated by rhizobial Nod factors *via* the CSSP (Dong et al., 2021; Quilbé et al., 2021). After nodule establishment, the plant exploits nodule transfer cells to sense the environmental nitrate status, to regulate plasticity of nodule development, by specifically expressing transporters of the NFP (NRT1/PTR FAMILY) (Wang Q. et al., 2020).

Some non-legume plants can also establish a symbiotic relationship with rhizobia, namely members of the genus *Parasponia*, also using the CSSP (van Velzen et al., 2018). However, other mechanisms are involved in the extracellular endophytic symbiotic relationship between non-legume plants and rhizobia. A typical case is that of the *non-expressor of pathogenesis-related (PR) genes 1* (*NPR1*), which is required to establish symbiotic relationship between barley and *Rhizobium radiobacter* (Kumar et al., 2021). In rice, bradyrhizobial strains use their quorum sensing system to sustain their symbiotic interaction with hosts (Pongdet et al., 2015; Cai et al., 2020). Non-rhizobial diazotrophic bacteria could also colonize rice, e.g., *Azoarcus olearius* which uses its flagella as a mediator of endophytic competence, or *Herbaspirillum seropedicae* (Pankievicz et al., 2021). Often, diazotrophic bacteria can fix air dinitrogen to produce ammonia that is usable by host plants (Peoples et al., 1995). The utilization of diazotrophs is a suitable and potential way for cereals to augment their nitrogen supply. An *Azoarcus* sp. strain was a typical diazotroph found in Karar grass plants (*Leptochloa fusca* L. Kunth), which can be co-cultured with rice (Hurek and Reinhold-Hurek, 2003). More recent work has demonstrated the utility of the diazotrophic bacterial associations of the grass *Setaria viridis* (e.g., with *Azospirillum brasilense* and *H. seropedicae*), as model systems for research aimed at enhancing biological nitrogen fixation for better nitrogen availability (Pankievicz et al., 2015, 2021).

Mycorrhizas are another typical model for the study of intracellular symbiotic interactions. The AM symbiosis is the most common mycorrhizal association, one that contributes to host-plant growth and development through nutrient absorption, in particular, of phosphate whose low solubility and mobility of phosphate in soil limits its accessibility to plants. In AM roots, plants make use of symbiotic interfaces provided by the fungal arbuscules or hyphal coils to take up the nutrients (Smith and Smith, 2011). The extraradical hyphal network explores the soil and takes up phosphates while inside the root, the plants employ mycorrhiza-specific phosphate transporters to transfer the phosphate released by the fungus into the root cortical cells; the fungal transporter that re-absorbs phosphate from the interfacial matrix acts as a sensor to control fungal growth and metabolism (Ferrol et al., 2019). The AM symbiosis can improve the growth of plants and enhance their tolerance against multiple stresses. In general, the symbiosis can be applied to phosphate-efficient farming systems aiming at sustainable agriculture (Ferrol et al., 2019).

Overall, the AM fungi colonize approximately 80% of land plants, while OM and ERM are specific for Orchidaceous and Ericoid hosts, respectively. To date, studies of ERM have sought basic knowledge of ERM symbiosis by using traditional methodologies, rendering details of its symbiotic mechanism that is mostly unknown (Vohník, 2020). By contrast, some symbiotic mechanisms for OM have been investigated. In their non-photosynthetic and photosynthetic stages, orchid plants respectively provide NH_4_^+^ and carbon to OM fungi to support fungal colonization (Dearnaley and Cameron, 2017). Orchid seeds harbor few reserves, so carbon sources, particularly lipids, provided by the fungus have to be harnessed for building a symbiotic structure, while the plant exports NH_4_^+^ (Bateman, 1995; Ghirardo et al., 2020). In the photosynthetic stage, the host accelerates fungal colonization by provision with sugars. As the fungus senesces and is digested in the plant cell, the host plant obtains nitrogen, phosphorus, and carbon from the hyphal coils (Dearnaley and Cameron, 2017). Research into the association between OM and orchid plants has focused mainly on fungal effects on plant growth, with few comprehensive metabolic and molecular mechanisms about their interaction actually studied yet. But based on the similar structure of nutrients exchange between AM and OM, some model of OM and their hosts could be proposed and investigated in depth with respect to interactive signaling pathways and the mechanisms of establishment of the symbiosis (Favre-Godal et al., 2020). Overall, these intracellular symbionts are crucial for the survival of several plant species, and will make a valuable contribution to broadening the biological and ecological strategies for use in agricultural applications in diverse and changing environments.

Some other plant–symbiotic fungi interactions were explored as well in recent years. It is generally known that *Serendipita indica* (formerly *Piriformospora indica*) can colonize a wide variety of terrestrial plants. The symbiotic relationship between *S. indica* and its host generally leads to a better crop productivity, strengthened tolerance against diverse stresses, and improved nutrient uptake and transport (Sharma and Varma, 2021). Therefore, the *S. indica*–host association can also serve as a reliable model system to explore the relevance of its interaction mechanism for crop improvements. Dark septate endophytes (DSEs) are a prominent part of the plant mutualistic symbiosis with plant roots, and are often capable of promoting tolerance of plants on certain abiotic stresses, such as low temperature (Chen et al., 2020), organic residues (He et al., 2020), and excess salinity (Yuan et al., 2021c). The DSEs show no host specificity and are capable of transferring nutrients from the soil into the plant cells. The benefits to hosts conferred by DSEs have emerged as an efficient strategy to augment the host growth and resistance given the low cost of culturing this type of microbe (Hidayat, 2019).

### Pathogenesis

Parasitism sits at one end of the parasitism–mutualism continuum, taking place when after its colonization, the microbe obtains resources from its host and in the process, causes damage (Newton et al., 2010; Drew et al., 2021). Along the continuum, if the symbiont can obtain benefits from the host at no cost in the absence of any enemies, then this microbe is on course toward a parasitic lifestyle (Vorburger and Gouskov, 2011). Notably, the degree of damage incurred by the host and the extent of resource acquisition could be intensified depending on a nutritious environment for pathogens and driven by a great selection pressure to propagate given the limited life span of the microbe (Rafaluk-Mohr, 2019; Lekberg et al., 2021). Once the microbe overcomes all the defense barriers in its host plant, thus, putting the host into disease state, it is usually called a pathogen.

Pathogens will actively destroy hosts for their own nutritious benefits and their interaction with plants is another type of plant–endophyte interaction. For example, *Pseudomonas syringae* is one of the best-studied bacterial species for its pathogenicity and the molecular mechanisms underlying the plant–microbe interactions with model plants (i.e., *Arabidopsis thaliana* and tomato); adding to its relevance, *P. syringae* infects almost all important economic crops and threatens global crop production (Xin and He, 2013; Xin et al., 2018). Type III secretion system (T3SS) effectors (T3Es) are the main mediators for the infection and immune evasion of *P. syringae*. Therefore, many studies have concentrated on the anti-pathogenic and pathogenic mechanisms of T3Es. Besides the eight “core” T3Es that were already discovered (Cunnac et al., 2011), it was found recently that plasmodesmata are also controlled by T3Es for expanding the spread of the bacterium in the host plant (Aung et al., 2020). Additionally, understanding of the T3SS regulatory network based on the transcriptional network has led to the identification of new candidates of T3Es (Fan et al., 2020; Shao et al., 2021), as well as the mechanism by which pathogenic growth is inhibited (Nobori et al., 2020; Xing et al., 2021). Because infection by *P. syringae* is affected by external environmental conditions, complete knowledge of the multidimensional nature of plant–*P. syringae*–environment–microbiota interactions is urgently needed as it will help to prevent diseases of crop plants (Xin et al., 2018).

Rice is a staple food crop worldwide and a model plant for basic research, but its yields and quality are always influenced by various diseases, such as bacterial leaf blight and leaf streak caused by *Xanthomonas oryzae* pv. *oryzae*, or rice blast caused by *Magnaporthe oryzae*. To confer a broad-spectrum and a durable resistance in this cereal plant, currently, the best way forward is to select among the inherent resistance genes of rice to breed disease-resistant cultivars. In this respect, many discoveries have been made recently, such as the RNase P protein subunit Rpp30 with tRNA processing (Li W. et al., 2021), executor R proteins’ (*Xa7* and *Xa23* with *EBE*_*Avrxa23*_) response to T3Es of pathogens (Chen X. et al., 2021; Wei et al., 2021), and *Bacterial Leaf Streak 1* (Ma Z. et al., 2021) which encodes a protein that acts against different pathogenic strains (Chen F. et al., 2021). As another economically important crop, wheat is susceptible to *Fusarium* head blight (Fhb) that is caused by several *Fusarium* strains. This disease severely limits wheat yields and causes the grains to accumulate deoxynivalenol, which poses problems for food safety and for the health of humans and animals (Bai and Shaner, 2004). To overcome the disease, broad-spectrum disease-resistant cultivars have been sought and bred by harnessing the *Fhb7* gene found in endophytic *Epichloë* fungi and located in the genome of the grass *Thinopyrum elongatum*, *via* distant hybridization (Wang H. et al., 2020). The establishment of a metabolite-molecular regulatory network affected by Fhb in wheat could further enhance our understanding of the mechanism of this disease (Su et al., 2021).

### Acquisition of Plant Stress Resistance From the Plant–Symbiont–Pathogen Relationship

In natural environments, certain microbiota assist plants in mitigating biotic and abiotic stress, mainly through two ways. The first is *via* horizontal gene transfer (HGT) (Li Y. et al., 2021). While transitioning from aquatic to terrestrial habitats, plants firstly underwent primary and secondary endosymbiosis involving HGT to withstand environmental challenges (Hiruma et al., 2016; Li et al., 2020). The HGT also took place outside the single evolutionary events of global significance: HGT The *macro2* domain gene originated from mycorrhizal fungi and helped plants to adapt early on to life on land (Wang S. et al., 2020). The *Fhb7* gene from endophytic *Epichloë* fungi, encoding a glutathione S-transferase for the detoxification of trichothecenes through de-epoxidation, has enhanced resistance of wheat to *Fusarium* head blight (Wang H. et al., 2020). On the other hand, the HGT of a syringopeptin synthetase homolog from *Pseudomonas* to *Burkholderia glumae*, the pathogen causing bacterial panicle blight, increased its virulence (Sai et al., 2021). The second is via a plant–symbiont–pathogen interaction that results in a continuous formation of novel defenses in hosts (Delaux and Schornack, 2021). In the course of evolution of host defense, plant–symbiont–pathogen interactions generated conserved gene modules, such as receptor-like kinases (Gong and Han, 2021), that are central to the plant innate immunity (Bentham et al., 2020). Notably, in the interaction with the fungal endophyte *Colletotrichum tofieldiae*, the fungus activates the plant’s PHT1 phosphate transporters which is beneficial under phosphorus-deficient conditions, while inducing plant defense by stimulating indole glucosinolate synthesis *via* the innate immune system (Hiruma et al., 2016). This would imply that symbiosis and pathogenesis can happen in the same interaction and also improved the host fitness.

The relationship between pathogens and hosts is not unlike an ‘arms race’ that reciprocally drives the immune system (mentioned above) and resistance to escalate because of the wide adaptability and heightened virulence of plant pathogens (Stringlis and Pieterse, 2021; Wang et al., 2021). However, plants could recruit some mutualistic microbes as partners to enhance their own immunity and to suppress pathogen infection. It seems then that microbes have adapted to be more cooperative with hosts for symbiosis and their co-existence confers better resistance to pathogens, which provides their host plants with superior growth and development over non-colonized ones (Batstone et al., 2020; McLaren and Callahan, 2020; Ma K.-W. et al., 2021). In other words, the plant, indeed, acts as a “microbial screening system” that distinguishes symbiotic microbes from pathogens.

### Comparison of the Interaction Process Between Symbiotic Microbes and Pathogens

The microbial invasion of plants is divided into two stages: the “recognition” phase, where the microorganism actively grows toward the plant (indicative of host recognition) and grows externally on the surface, and the “colonization” phase, where the microbe invades the plant (Mukherjee et al., 2012; Vahabi et al., 2015b). The microbe must pass through four different host barriers: plant microbiota, physical cell barrier, and two plant immune systems, i.e., innate or microbe/pathogen-associated molecular patterns (M/PAMPs)-triggered immunity (PTI) and effector-triggered immunity (ETI), for a successful invasion to occur (Hacquard et al., 2017).

#### Recognition Phase

Plants possess a general stress response involving “core immunity responses” genes (Bjornson et al., 2021; Cole and Tringe, 2021), as well as the general “non-self” response (Maier et al., 2021). Plants possess various PRRs (pattern recognition receptors) that recognize M/PAMP ligands and initiate immune reactions. In response to microbial infection, plants can induce disease resistance genes that recognize pathogen-specific ligands. Both pathogens and symbionts can be recognized by PRRs because the M/PAMPs are not specific to pathogens; they include fragments of essential microbial protein and glycans.

To avoid recognition by the host plant followed by immune response, pathogens and symbionts have the evolved complex extracellular invasion strategies. Due to the similarity of pathogen and symbiont genomes (Reinhardt et al., 2021), common extracellular strategies exist between them, and they can be divided into three categories: avoiding accumulation of MAMP precursors, reducing hydrolytic MAMP release, and preventing MAMP perception (Buscaill and van der Hoorn, 2021), which can involve different microbial effectors. The effectors include the LysM effector (Sánchez-Vallet et al., 2020), salicylate hydroxylase (e.g., wheat and *Fusarium graminearum*) (Qi et al., 2019), cell wall degrading enzymes (CWDE) (*A. thaliana* and *S. indica*) (Hacquard et al., 2016), T3Es (e.g., *A. thaliana* and *P. syringae*), and EPS (e.g., the few basidiomycete-based lichen symbioses) (Wanke et al., 2021). For example, *X. oryzae* pv. *oryzae* secretes some effectors to upregulate the *SWEET* genes of rice so as to provide it with nutrition, especially AvrXa7 and AvrXa23 (Vera Cruz et al., 2000; Gupta, 2020), but they can be detected by Xa7 and Xa23 with EBE_Avrxa23_ in wild rice (Chen X. et al., 2021; Wei et al., 2021). Effector proteins are also secreted by oomycetes into the host to promote infection and suppress the immune system. This is exemplified by the “RxLR” motif family of effectors from *Hyaloperonospora arabidopsidis*, one of whose members, HaRxL21, interacts with the co-repressor Topless (Harvey et al., 2020), and an AM fungus whose secretion contains the LysM effector to subvert the chitin-triggered immunity response by the host plant (Zeng et al., 2020).

Furthermore, symbionts have developed various strategies to let their potential hosts better distinguish them from pathogens during the recognition phase. For example, lipochitooligosaccharide Nod Factors are perceived by legumes (Radutoiu et al., 2003; Bozsoki et al., 2020). In rice, short-chained chitotetraose triggers symbiotic signal transduction with the symbiotic complex receptor MYR1–CERK1, and this suppresses the formation of the CEBiP-CERK1 heteromer (Chiu and Paszkowski, 2021; Zhang et al., 2021). Besides, symbionts are also capable of colonizing hosts while overcoming the response to damage-associated molecular patterns (DAMPs) and MAMPs, while a response against pathogens is possible in the presence of non-pathogenic microbes (Zhou et al., 2020). Also, symbionts could induce jasmonic acid (JA) and suppress salicylic acid (SA) formation to induced systemic resistance (ISR), whereas pathogens typically enhance the SA biosynthesis to mediate systemic acquired resistance (SAR) in plants (Martínez-Medina et al., 2017).

#### Colonization Phase

After overcoming a plant’s microbiota and physical barriers, microbes will move across the different cell layers of the host and this movement elicits the response of cell-layer-specific programs of the plant (Fröschel et al., 2021). At this point in the colonization process, the growth of some pathogens will be suppressed by the endodermal barrier, while some will utilize molecular decoys to protect themselves (Ma Z. et al., 2017; Xia et al., 2020) and to penetrate the adjacent cell regions by regulating the pore size of plasmodesmata (Huang et al., 2019). Meanwhile, some pathogens inevitably trigger the NLR-mediated immunity involving a PTI-ETI crosstalk (Ngou et al., 2021; Yuan et al., 2021a,b). Finally, those that slip past and evade the immune response of the host will colonize it further to cause a disease.

For arbuscular mycorrhizal fungi (AMF), two receptor-like kinases called arbuscular receptor-like kinase 1 (ARK1) and ARK2 are employed to maintain the symbiotic interaction (Montero et al., 2021). In turn, plants employ a liquid-liquid phase separation to compartmentalize cellular activities in membrane-less organelles, protecting their microbial symbionts from the immune-response (Shin and Brangwynne, 2017; Huang et al., 2021). Plant autoimmunity and other physiological responses occur in the nucleus, leaving symbionts able to dwell safely in the intracellular matrix. Meanwhile, AMF are separated from the plant cytoplasm by a specialized host-derived membrane, which represents the main interface facilitating the bidirectional exchange of nutrients and information. The biosynthesis of this periarbuscular membrane is controlled by a gene called *glucosamine inositol phosphorylceramide transferase 1* (Moore et al., 2021). The endophytic fungus *S. indica* secretes specific proteins to improve its access to micronutrients and to influence oxidative stress and reactive oxygen homeostasis to support the colonization of the host plant (Nostadt et al., 2020). A bacterial endophyte can utilize the hyphae of a fungal pathogen to gain access from the soil to plant roots, thereby protecting the host from infection (Palmieri et al., 2020), while others may use Type II secretion systems to modulate PTI to colonize the host (Teixeira et al., 2021). Symbiotic and pathogenic microbes do share some similar characteristics, but they also differ in certain ways; hence, plant–microbe interaction is a complicated phenomenon that requires multi-omics technologies for its in-depth research.

## Background and Progress in the Development of Multi-Omics Technology

Omics can be used to perform a global analysis of biological samples in a high-throughput manner. In the early phase of molecular biology research, the invention of Sanger sequencing was a revolutionary breakthrough. However, it is only suitable and accurate for the study of short sequences. Since then, it has been improved to enable the sequencing of the first human genome, making it a great contribution to the treatment of medical diseases. However, the cost and time spent when using Sanger sequencing were enormous, which limits its accessibility and use by most studies. In stark contrast, next generation sequencing (NGS) allows for genetic sequencing in a high-throughput and less expensive way, thereby enabling the transcriptomes and genome of a species to be studied in detail. Nevertheless, the assembled sequence length is not sufficient to resolve the whole genome’s information. The development of third-generation sequencing, which is capable of long-read sequencing, is therefore a giant step forward in overcoming that problem (van Dijk et al., 2018). The continuous development of sequencing technology facilitates the discovery of new genes and makes the construction of regulatory networks of known genes possible. Meanwhile, other omics methodologies have been developed and are being used across multiple fields of research. Among these, metabolomics is a useful tool to identify and to quantify the full set of metabolites in an organism, which could be then related to its phenotypic features under dynamic circumstances.

Single omics provides a view of the biological processes limited to a single level and the analysis is, nonetheless, limited to correlations, which may generally conclude with consequential variation rather than causative ones. We now need to acquire global information integrated *via* several single omics technologies, which is called the multi-omics approach. For example, an interactome, generated by at least genomics and transcriptomics, is essential for studying mechanisms related to plant–microbe interactions. Genomic sequencing provides the pre-requisite genomic information, while the transcriptome findings link the gene function with specific conditions, and the metabolome information correlates the vast array of metabolites involved in multiple pathways, which in turn reflects the occurrence of corresponding gene expression events and their levels (Qin et al., 2016). When combined with other biological data, a genetic, protein, and metabolite multi-omics study is a complementary approach that can be utilized to decipher the individual signal molecules, proteins, genes, and gene regulation cascades, and their relationship in gene networks/pathways, thereby deepening our understanding of, e.g., the microbe-mediated mitigation of stresses in plants (Yin et al., 2014; Luan et al., 2015).

### Transcriptomics Technology

**FIGURE 1:**
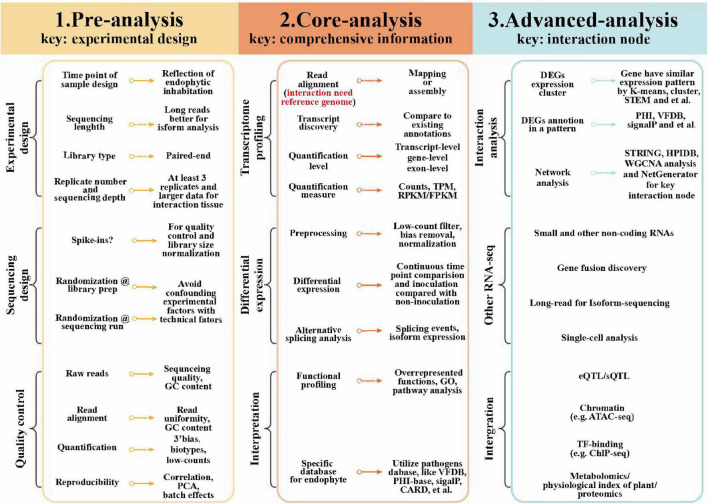
Transcriptomic workflow for plant–endophyte interaction studies. DEG, differential expressed gene; ChIP-seq, chromatin immunoprecipitation sequencing; eQTL, expression quantitative loci; FPKM, fragments per kilobase of exon model per million mapped reads; GSEA, gene set enrichment analysis; PCA, principal component analysis; PHI, pathogen host interactions; RPKM, reads per kilobase of exon model per million reads; sQTL, splicing quantitative trait loci; TF transcription factor; TPM, transcripts per million; HPIDB, host–pathogen interactions database; VFDB, virulence factors database; STRING, search tool for the retrieval of interacting genes; and WGCNA, weighted correlation network analysis.

The sequencing of mRNA is a useful approach to study the responses of different plants in relation to their endophytes, by generating the transcriptome-level information (Sheibani-Tezerji et al., 2015). The transcriptome analysis involves comparing endophyte-free (E−) and endophyte-inhabited (E+) plants to understand the basis of endophyte-mediated host responses. An adjustable transcriptomic workflow suitable for the study of plant–microbe interactions is summarized in Figure 1 (Conesa et al., 2016). Transcriptional profiling can be done using various methods, including microarray analysis, SOLiD-SAGE, and RNA sequencing (RNA-seq) (Table 1). In particular, RNA-seq has many recognized strengths: it allows differential expression analysis under different conditions, simultaneous detection of microbial and host gene expression, and the discovery of novel RNA candidates. Microarray analysis and SOLiD-SAGE are useful tools for conducting a differential gene expression analysis. With the emergence of third-generation sequencing, one can readily perform a global transcriptome analysis and obtain a host-specialized transcriptome, even when using a single-cell RNA sequencing and a spatial transcriptome to accurately study the transcriptional reaction of each cell. Through transcriptomic analysis, we can now uncover the dynamics and regulation of actively transcribed genes, thus, offering considerable advantages *vis-à-vis* a genomic analysis alone (Levy et al., 2018).

**TABLE 1:** Overview of the plant–endophyte interaction at transcriptomic level.

No.	Hosts	Endophytes	Sample preparation	Sequencing platform	Differentially expressed genes		References
					Up-regulation	Down-regulation	
1	*Arabidopsis thaliana*	*Serendipita indica*	Roots and shoots of seedlings	*Arabidopsis* Genome Array ATH1	Stress- and defense-related genes including plant hormones and signaling, antioxidants and secondary metabolism at early stage; growth plant hormones and signaling, primary metabolism at later stage		Vahabi et al., 2015a,b
2	*Arabidopsis thaliana*	*Paecilomyces variotii*	Leaves	–	Auxin genes at low concentrations, SA biosynthesis and signaling pathways at high concentrations		Lu et al., 2019
3	*Arabidopsis thaliana*	*Trichoderma asperelloides* T203	Roots	Agilent *Arabidopsis* Gene Expression Microarrays	*WRKY18*, *WRKY40*, *WRKY60*, *WRKY41*, *WRKY53*, *WRKY 55*, *JAZ*, *FMO1*, *PAD3*, *CYP7A13*, ACC deaminase, ET signaling pathway, *MDAR*, *APX1*, *GST*		Brotman et al., 2013
4	*Zea mays*	*Trichoderma virens*		NovaSeq 6000	JA biosynthesis at recognition, SA degrading, cell wall degrading and transcription factors and signal transduction genes at colonization	SA degrading and second metabolites genes at recognition, JA biosynthesis at colonization	Malinich et al., 2019
5	Rice	*Harpophora oryzae*	Roots	Illumina HiSeq 2000	Glycolysis and the TCA cycle	Shikimate and lignin biosynthesis pathways	Xu et al., 2015
6	Rice	*Trichoderma asperellum*	Seedling leaves	HiSeq machines	Genes related to plant growth enhancement, physiological functioning, photosynthesis, RNA activity, stomatal activity, and root development		Doni et al., 2019
7	*Dendrobium nobile*	*Mycena* sp.	1 week-old and 9 week-old stem	Illumina HiSeq 4000	*petF*, *SUS*, *bcsA*, *glgA*, *PMM* and *GPMM AACT*, *MVD*, *PMK* and *TPS21* at 9 weeks, post-modification enzymes genes	PGK, PFK, PDHB, CS and SDHA genes	Li et al., 2017a,b
8	*Salvia miltiorrhiza*	Endophytic fungi U104	Whole plant	Illumina HiSeq 2000	Genes in the tanshinone biosynthesis pathway: *DXS*, *DXS2*, *DXR*, *AACT*, *MK*, *PMK*, *GGPPS2*, *GPPS*, *KSL*, *IDI*, *IPII, FDPS*, *HMGR3* and *CPS*.		Jiang Y. et al., 2019
9	*Atractylodes lancea*	*Gilmaniella* sp. AL12	Shoot tissue	Illumina HiSeq™ 4000	Genes related to primary metabolism and involved in terpene skeleton biosynthesis, and upregulated genes annotated as β-farnesene synthase and β-caryophyllene synthase.		Yuan et al., 2019
10	*Anoectochilus roxburghii*	*Ceratobasidium* sp. AR2	The whole plantlet	Illumina HiSeq 2000	Gene in flavonoid biosynthesis: *PAL*, *4CL*, *CHS*, *GT*, and *RT*		Zhang et al., 2020
11	Barley	*Serendipita vermifera*	100–200 mg ground root sample	HiSeq 2500 system	Genes involved in hydrolytic enzymes, detoxification and redox homeostasis		Sarkar et al., 2019
12	*Lolium arundinaceum*	*Epichloë coenophiala*	Leaf blade, crown; root; pseudostem	Illumina HiSeq 2500	WRKY transcription factors	Genes involved in response to chitin, respiratory burst during defense response and intracellular signal transduction	Dinkins et al., 2017
13	Barley (*Hordeum vulgare* L.)	*Serendipita indica*	Leaf samples	Barley PGRC2 13 K cDNA array	*AUX1*, *AXR1*, *USP*, ACC oxidase, ET signal molecule, *AP2*/*ERF*, *TAX*, *APX*, *VTC2*, peroxidase, HSP70	MBF1A, ACC oxidase, HV22D, ABA deficient 1, bHLH	Ghaffari et al., 2016
14	*Zea mays* cv. *Jixiang 1*	*Serendipita indica*	Roots	Illumina HiSeq	Tubulin, kinesin; SA, ABA, CTK, and auxin biosynthesis and signaling pathways; cutin, suberin, and wax biosynthesis		Zhang et al., 2018
15	Tall fescue (P12, P27, P46, P12)	*Epichloë coenophiala* (CTE, NTE19, FaTG4)	Pseudostems	Illumina HiSeq 2000 Illumina HiSeq 2500	Speculation: repression of some genes involved in fungal and defense response and priming of some genes that may enhance drought tolerance		Dinkins et al., 2019
16	*Achnatherum inebrians*	*Epichloë gansuensis*	Seeds	Illumina/Solexa sequencing system	Genes involved in unsaturated fatty acids, alkaloids biosynthesis and ROS scavenging, HSPs		Chen et al., 2016
17	*Arundinella bengalensis*	*Exophiala pisciphila*	Hyphae	e Illumina Hiseq 2000	Metal ion binding and transportation, organic acid metabolism and transportation, ROS scavenging, redox homeostasis, transcription factors production, sulfate assimilation, DNA repair and cell wall integrity maintenance		Zhao et al., 2015
18	*Arachis hypogaea*	*Metarhizium anisopliae*	Plant medullar tissue	Illumina Hiseq 2500	ERF branch of JA signaling pathway	Hypersensitive response and negative regulation of defense	Hao et al., 2017
19	*Lolium perenne*	*Epichloë festucae*	Leaves (seven sections)	Illumina	Genes involved in hyphal growth in growth host tissues, synthesizing antiherbivore compounds in mature plant tissues, hormone biosynthesis and perception as well as stress and pathogen resistance	Photosynthesis genes	Schmid et al., 2017
20	Rice	*Trichoderma harzianum* Th-56	Leaf samples	51 K Affymetrix gene chip	*DREB*, superoxide dismutase Aquaporin, Dehydrin		Pandey et al., 2016
21	Rice	*Phomopsis liquidambari*	Mycelium	Illumina HiSeq™ 2000	Genes from amino acids metabolism, carbohydrate metabolism, fatty acid biosynthesis, secondary metabolism, and terpenoid and steroid biosynthesis		Zhou et al., 2017
22	*Miscanthus sinensis*	*Herbaspirillum frisingense* GSF30^T^	Roots and shoots	Illumina	Photosynthesis- and JA-related genes at two stages	Ascorbate and glutathione, flavonoids ET-related at two stages.	Straub et al., 2013
23	Banana (*Musa acuminata*)	*Pseudomonas fluorescens* Ps006;*Bacillus amyloliquefaciens* Bs006;	Whole seedlings	Illumina Hi Scan SQ™	L-ascorbate oxidase gene, palmitoyl-acyl carrier protein thioesterase, wound-induced protein, CYTC, Cyt P450, *AOX2* under two endophytes treatment		Gamez et al., 2019
24	Rice	*Herbaspirillum seropedicae*	Roots	SOLiD4 sequencer	The synthesis and efflux of phytosiderophores (PS) and transport of PS-iron complexes	Genes related to plant defense, plant disease resistance, flavonoid and isoprenoid synthesis	Brusamarello-Santos et al., 2019
25	*Arabidopsis thaliana*	SSTP endophytic bacteria	Roots and leaves	–	*AKT1*, *GORK*, *KUP6*, *SOS1*, and *SOS3* in shoots, proline and trehalose genes	HAK5, SKOR, and HKT1 in roots	Eida et al., 2019
26	Rice	*Burkholderia kururiensis* M130	2 × 10^9^ bacteria cells	Illumina HiSeq 2000	Motility, chemotaxis, and adhesion related to membrane transporters and secretion systems		Coutinho et al., 2015
27	*Arabidopsis thaliana*	*Bacillus amyloliquefaciens* FZB42	Shoot tissues	HiSeq. 2000 system	Gene related to ROS scavenging, photosynthesis, auxin, ET/JA signaling and ABA-independent pathways, Na^+^ translocation, and osmoprotectant synthesis		Liu et al., 2017
28	*Arabidopsis thaliana*	*Pseudomonas chlororaphis* O6	Aerial parts of the plants	Affymetrix GeneChips	Plant disease resistance	Drought signaling response genes	Cho et al., 2013
29	Potato	*Burkholderia phytofirmans* PsJN	Frozen plant material, pure bacterial cultures	Illumina HiSeq 2000	Extracytoplasmatic function 164/429/866, oxidative phosphorylation, ROS detoxification genes, transcription regulation, cellular homeostasis, and cell redox homeostasis	Glutathione metabolism, a two-component system, and the pentose phosphate pathway	Sheibani-Tezerji et al., 2015
30	Rice	*Azoarcus* sp. BH72	Bacteria cells	oligonucleotide-coated microarray	Genes involved in bacterial motility and endophytic root colonization bacterial colonization factors		Sarkar et al., 2017
31	Potato	*Bacillus mycoides* EC18	Bacteria cells	–	Genes involved in organic substance metabolism, oxidative reduction, transmembrane transport, membrane proteins, transcriptional regulators, amino acid metabolism and biosynthesis		Yi et al., 2017
32	–	*Herbaspirillum seropedicae*	Bacteria cells	ION Proton semiconductor sequencer	Genes coding for outer membrane: TonB-dependent receptors, inner membrane: high affinity permease and iron-regulated membrane protein, and EPS biosynthesis		Trovero et al., 2018
33	*Triticum aestivum*	*Herbaspirillum seropedicae*	Root attached and planktonic bacteria	SOLiD 4 platform sequencing	Specific adhesins and cell wall remodeling, plant growth promotion nitrogen-fixation, polyhydroxybutyrate synthesis, and ABC transporter (up- or down-regulated are not mentioned)		Pankievicz et al., 2016
34	Sugarcane	*Gluconacetobacter diazotrophicus*	Root and shoot samples	Illumina HiSeq 2000	ABA biosynthesis and signal transduction activation in shoots	ABA, ET and auxin biosynthesis and signal transduction repression in roots; ET and auxin biosynthesis and signal transduction repression in shoots	Vargas et al., 2014
35	*Limonium sinense*	*Glutamicibacter halophytocola*	Leaves	Illumina HiSeq 2500	Pathways related to phenylpropanoid and flavonoid biosynthesis and ion transport and plant hormone metabolism and signal transduction		Qin S. et al., 2018

*No. 1–21 related with endophytic fungus; No. 22–35 related with endophytic bacterium.*

Despite that, genome-based studies constitute a pivotal foundation for performing successful transcriptomic studies. Thus, pursuing genome and transcriptome analyses in tandem is more helpful for decoding the nature of plant–endophyte interactions (Kaul et al., 2016).

### Metabolomics

Knowledge of plant–microbe interactions has increased substantially in recent years; however, the chemical communication leading to the priming is not yet well understood. Metabolomics, an array of advanced bioanalytical techniques in conjunction with chemometrics and bioinformatics tools, enables the characterization of perturbations to the metabolomes of interacting organisms (Mhlongo et al., 2018), thus providing a complement for other “-omics” techniques. Metabolomics can facilitate a greater understanding of the independent metabolisms of the plant and its endophyte, as well as the metabolic crosstalk that underpins the interactome (Jorge et al., 2016; Ofaim et al., 2017).

**FIGURE 2:**
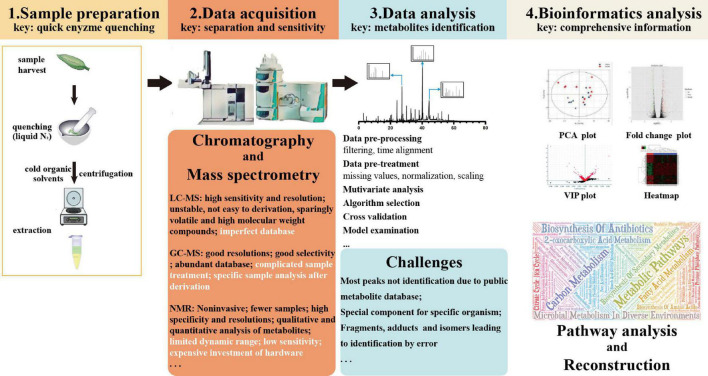
Flowchart for plant metabolomic studies. N2, nitrogen; LC-MS, liquid chromatography-mass spectrometry; GC-MS, chromatography-mass spectrometry; NMR, nuclear magnetic resonance; PCA, principal component analysis; and VIP, variable importance for the projection.

Several techniques are available to characterize metabolites extracted from fungi. An adjustable metabolomics flowchart includes sample preparation, data acquisition, data mining and analysis, statistical modeling, signatory biomarkers, and biochemical interpretation (Figure 2; Mhlongo et al., 2018). Different researchers have used different methods for metabolite profiling (Table 2). To separate and identify compounds, many detection techniques are widely used, such as column chromatography (CC), flash chromatography (FC), Fourier transform infrared spectroscopy (FTIR), thin layer chromatography (TLC), gas chromatography–mass spectrometry (GC-MS), liquid chromatography–mass spectrometry (LC-MS), LC-MS/MS, liquid chromatography-ultraviolet, and visible spectrum or diode array detection [LC-UV (DAD)], different types of high-performance liquid chromatography (HPLC), gas–liquid chromatography, nuclear magnetic resonance (NMR) imaging, and liquid chromatography/time of flight-mass spectrometry (LC-TOF/MS) (Khoomrung et al., 2017; Maheshwari, 2017; Xie et al., 2017). When traditional MS is not able to accurately detect spatial-temporal occurrences of metabolites, the spatial metabolome may be exploited based on desorption electrospray ionization-imaging mass spectrometry (DESI-IMS) and matrix-assisted laser desorption/ionization-IMS (MALDI-IMS), as well as using an air flow-assisted desorption electrospray ionization mass spectrometry imaging (AFADESI-MSI) (He et al., 2018).

**TABLE 2:** Overview of the plant–endophyte interaction at metabolomic level.

Host status	No. Hosts	Endophytes	Sample preparation	Detection platform	Differentially expressed metabolites		Metabolite effect	References
					Increase	Decrease		
Plant growth promotion	1 Cucumber	*Paecilomyces formosus* LHL10	50 mL culture medium 0.5 g freeze-dried plant samples	HPLC, GC/MS-SIM	Proline antioxidants GA and IAA,	ABA	Facilitate plant growth modulate stress	Khan et al., 2012
	2 Maize	*Fusarium culmorum* Pz11	Exudates of fungus culture and roots	HPLC, LC-ESI-MS/MS	IAA, flavonoids and sugars		Enhance plant growth	Mehmood et al., 2019
	3 *Arabidopsis thaliana*	*Trichoderma virens*	Fungal cultures	GC-MS	IAA and indolic compound		Facilitate plant growth	Angel Contreras-Cornejo et al., 2009
	4 Tomato and oilseed rape	*Trichoderma* strains	Fungal cultures	TLC, MS	Auxin-like effect matters		Facilitate plant growth	Vinale et al., 2008
	5 *Parachlorella kessleri-I*	*Serendipita indica*	Fungal pellets, plant cultures	GC-MS, HPLC, LC-MS	Succinate, oxo-propanoate, l-alanine, glutamate, acetate and 1,2 propanediol, hydroxy butane, fatty acid methyl ester		Influence plant growth and lipid profile	Bhatnagar et al., 2019
	6 Tall fescue	*Epichloë* *coenophiala*	Root exudates	GC-TOF MS	Lipids, carbohydrates and carboxylic acids		Affect Plant biomass and root exudate	Guo et al., 2015
	7 Sugarcane	Diazotrophic endophytes	Microbial cultures	HPLC	Amounts of amino acids	ET	Promote plant growth	de Santi Ferrara et al., 2012
	8 Rice	*Bacillus amyloliquefaciens* RWL-1	Bacteria culture filtrates	GC-MS/SIM, LC–MS/MS, quadrupole TOF	IAA, glutamic and alanine, antioxidant properties, proteases, metabolism enzymes, ribosomal proteins, antioxidant proteins, chaperones, and heat shock proteins	ABA	Promote plant growth	Shahzad et al., 2019
Host-metabolite accumulation	9 Grape	Foliar endophytic fungal strains	10 mg of grape callus powder	HPLC	Novel metabolites, reducing sugar, total flavonoids, total phenols, trans-resveratrol, activities of phenylalanine ammonia-lyase		Shape grape qualities	Yang et al., 2016; Huang et al., 2018
	10 Wheat	*Trichoderma atroviride*, *Rhizoglomus irregulare*	50 mL freeze dried root exudates	UHPLC/QTOF-MS; GC/MS	Lipids (sterols and membrane lipids), phenolic compounds and terpenoids, siderophores and chelating acids, derivatives of amino acids and phytohormones	Support of biostimulant effect		Lucini et al., 2019
	11 *Bixa orellana*	*Botryosphaeria mamane*	The broth crude extracts	UHPLC-HRMS	Isocoumarins, dipeptides, benzopyranoids, aliphatics, and trichothecenes	Phosphoethanolamins, phosphatidylserines, phosphatidylcholines, lanostane triterpenoids, diterpenoids, and several hybrid peptides	/	Triastuti et al., 2019
	12 *Hypericum*	*Thielavia subthermophila*, *Fusarium oxysporum*, *Trichoderma crissum, Serendipita indica*	50 mg of air-dried shoots	HPLC	Anthraquinones phloroglucinols, hydroxycinnamic acids, and flavonoids		/	Bálintová et al., 2019
	13 *Centella asiatica*	*Serendipita indica*	Plant powder	HPLC	asiaticoside		/	Jisha et al., 2018
	14 Grape	*Bacillus amyloliquefaciens, Pseudomonas fluorescens*	1 mL of the bacterial culture 0.5 g roots powder samples	UPLC-MS/MS	Melatonin secretion, 5-hydroxytryptophan, serotonin, and *N*-acetylserotonin	Tryptamine and serotonin	Counteract the adverse effects	Jiao et al., 2016; Ma Y. et al., 2017
Abiotic stress resistance enhancement	15 Barley	*Serendipita indica*	100 mg of freeze-dried leaf sample	DIC-QQQ; GC-MS	Soluble sugars, amino acids, and organic acids	Mitigate oxidative stress		Ghaffari et al., 2019
	16 Wheat	*Acremonium sclerotigenum, Sarocladium implicatum*	50 μg of dry leaves	UPLC-QTOF MS	Proline by *S. implicatum*	Levels of stress damage markers and reduced accumulation of stress-adaptation metabolites (ABA, jasmonic isoleucine and lipid peroxidation, malondialdehyde, caffeic, and ferulic acids)	Reduce levels of water-limited stress	Llorens et al., 2019
	17 Tall fescue	*Neotyphodium coenophialum*	100 mg of ground lyophilized plant material	HPLC, LC-MS	Free glucose, fructose, trehalose, sugar alcohols, proline, and glutamic acid in shoots and roots; the fungal metabolites, mannitol, and loline alkaloids		Aids plant to survive and recovery	Nagabhyru et al., 2013
	18 *Cephalotaxus harringtonia*	*Paraconiothyrium variabile*	40 g leaves	HPLC; LC/MS; LC/MS-MS; QTOFMS	Glycosylated flavonoids, deglycosylated flavonoids (increased or decreased are not mentioned)		Hyphal growth	Tian et al., 2014
	19 *Cirsium arvense*	*Chaetomium cochlioides*	0.1 g leaves; 100–200 mg endophyte cultures	UPLC; QTOFMS;	Several novel oxylipin metabolites		Hypersensitive reaction	Hartley et al., 2015
	20 *Festuca pratensis* Huds. *Lolium perenne* L.	*Epichloe uncinata*	1 g ground sample	LC-MS/MS; LC-TOF/MS; LC-QTRAP/MS	*N*-formylloline, *N*-acetylcholine		Protection against pests	Adhikari et al., 2016
	21 Tomato	*Trichoderma harzianum*	10 mg of ground tomato	QTOF LC-MS/MS	Steroidal glycoalkaloid, lycoperoside, cholesterol		Activate chemical defenses	Manganiello et al., 2018
Abiotic stress resistance enhancement	22 *Kadsura angustifolia*, wheat bran	*Umbelopsis dimorpha* SWUKD3.1410	6 g wet *K. angustifolia* stems and roots and wheat bran; 6 g of sterilized wet solid media (*K. angustifolia*) 100 g wet *K. angustifolia*	TLC; HPLC; NMR; HR-ESI-MS	Aromatic compounds schitriterpenoid, lignan, sterol, trinorsesquiterpenoid, sesquiterpenoid, and monoterpene	provide the plant defense for healthy growth		Qin D. et al., 2018
	23 Sugarcane	*Herbaspirillum seropedicae, Gluconacetobacter diazotrophicus*	20 mg of fresh weight leaves	GS-TOF-MS	Heptuloses, riboses, glucuronate, amino sugars, lipids, amino acids and phytosterol (mevalonate pathway), by humic acids and endophytes		Cellular redox	Aguiar et al., 2018
	24 *Alnus glutinosa*	*Frankia*	150 mg of dried powder (nodule and associated-root)	GC/EI-QQQ, HPLC/DAD, HPLC/DAD/ESI-Q-TOF	Amino acids, SOA 16 (glucose) and secondary metabolites, especially 5-*O*-β-D-xylopyranoside		Plant defense and signal induction	Anne-Emmanuelle et al., 2017
	25 *Salsola imbricata*	*Streptomyces* sp. (EA65, EA67)	5 mL of a bacterial culture	UPLC; TOF/Q-TOF MS	Antifungal metabolites such as Sulfamerazine, Sulfamethoxypyridazine, and Dimetridazole		Antibiotics of antagonistic bacterial	Bibi et al., 2018
	26 *Sambucus nigra*	Endophytic communities	100 mg of dried powder (leaves and flowers)	LC-MS	Pyrogallol appeared in the floral epiphytic extracts, ketoglutarate-synthesis pathway in foliar epiphytic extracts	Lactate, citraconic acid, acetyl-CoA, isoleucine, and several secondary compounds in foliar epiphytic extracts	Amino acid biosynthesis	Gargallo-Garriga et al., 2016

*No. 1–6, 9–13, and 15–22 related with endophytic fungus; No.7, 8, 14, and 23–26 related with endophytic bacterium.*

## Transcriptomics and Metabolomics Applied to Plant–Endophyte Interactions

Numerous reports have elucidated how endophytes can promote plant growth, accelerate metabolism, and strengthen stress resistance in hosts. However, plant–endophyte interactions require complex and involve various response mechanisms. Nevertheless, it is now feasible to glean in-depth knowledge of these mechanisms by implementing transcriptomic and metabolomic techniques.

### The Plant–Endophyte Interaction: From Inhabitation to Stress Resistance

**FIGURE 3:**
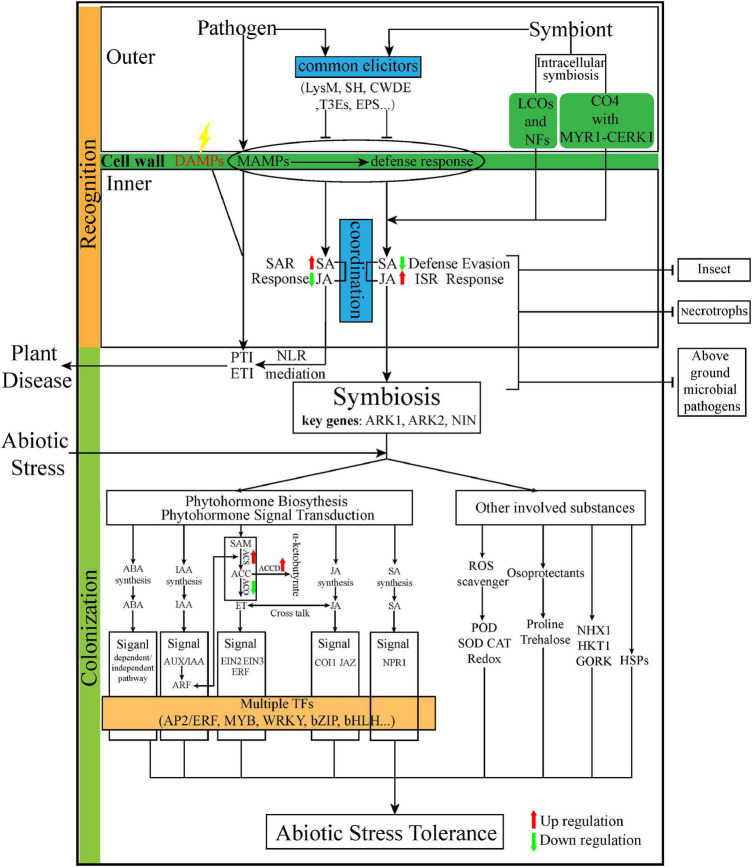
The summarization of the interaction from infection to tolerance of diverse abiotic stress. Red dotted arrow represents that elicitors/effector are one step ahead of microbes. Red and green arrows represent up and downregulation of genes or compounds, respectively. LysM, lysine motifs; SH, salicylate hydroxylase; CWDE, cell wall degrading enzymes; T3Es, Type III secretion system effectors; NFs, Nod factors; LCOs, lipochitooligosaccharides; CO4, short-chain chitotetraose; CERK1, chitin elicitor receptor kinase 1; SAR, systemic acquired resistance; ISR, induced systemic resistance; NLR, nucleotide-binding leucine-rich repeat receptor; PTI, pathogen-associated molecular patterns (PAMPs)-triggered immunity; ETI, effector-triggered immunity; ARK, arbuscular receptor-like kinase; NIN, nodule inception transcript factor; SA, salicylic acid; JA, jasmonic acid; ABA, abscisic acid; IAA, indole-3-acetic acid; ET, ethylene; SAM, S-adenosyl methionine; ACC, 1-aminocyclopropane-1-carboxylate; ACS, ACC synthase; ACO, ACC oxidase; ACCD, ACC deaminase;NPR1, non-expressor of pathogenesis-related genes 1 ROS, reactive oxygen species; POD, peroxidase; SOD, superoxide dismutase; CAT, catalase; NHX1, Na+/H+ antiporter; HKT1: high-affinity K+ transporter 1; GORK, gated outwardly rectifying K+ channel; HSP, heat shocked protein; and TFs, Transcriptional Factors.

Transcriptomic studies have addressed different aspects of the plant–endophyte interaction, spanning endophyte inhabitation (recognition and colonization) to stress resistance (Figure 3).

#### Recognition Phase

During the recognition phase, endophytic microbes exhibit different mechanisms of interaction with hosts to evade host defenses before colonization. For example, a number of fungal endophytes mask chitin to forestall further defensive reactions (Sánchez-Vallet et al., 2013, 2015), to repress plant SA production (Navarro-Meléndez and Heil, 2014), and to promote JA biosynthesis (Straub et al., 2013; Pinski et al., 2019). Some endophytes employ miRNA-targeted regulating pathways to turn off the defense system in the host plant (Plett and Martin, 2018). In the recognition phase, compounds secreted by endophytes enter the host as messengers before microbial entry, to promote plant growth (Jiang C. H. et al., 2019; Lu et al., 2019), and they activate both stress and defense responses against pathogens (Vahabi et al., 2015b; Lu et al., 2019). In a study of *Arabidopsis* inoculated with *S. indica*, after this physical contact, the fold-change in the JA content was much lower than that of SA, reversing the situation that existed before the physical contact (Vahabi et al., 2015a,b). A recent study of the *Trichoderma virens*–maize interaction (Malinich et al., 2019) showed that *T. virens* reduced a plant SA formation. Meanwhile, the reduced SA formation prevented the production of catechol from SA, which increased the plant susceptibility to pathogen attack (Ambrose et al., 2015), while JA biosynthesis genes were upregulated to stimulate the establishment of ISR against necrotrophs. The SA levels in colonized plants, however, were not significantly different from those in non-colonized plants (Ambrose et al., 2015). That is, the physical contact between the two symbionts might not result in the increase of SA levels, but it may nonetheless suffice to establish a systemic defense, depending on the coordination between SA- and ethylene/JA-induced plant defenses (Tsuda et al., 2009; Hillmer et al., 2017; Constantin et al., 2019).

#### Colonization Phase

After overcoming a plant’s competing microbiota and physical barriers, endophytes may adopt different strategies to prevent themselves from being targeted by host defense responses. Endophytic bacteria format their own MAMPs (Vandenkoornhuyse et al., 2015) or produce enzymes in response to the immune system of their host (Trdá et al., 2015), while fungal endophytes rely on secreting chitin deacetylase or fungal lectins (Lahrmann and Zuccaro, 2012). In contrast to pathogenic microbes which elicit SAR through SA signaling, endophytes regulate JA/ET signaling to induce ISR which enhances the tolerance of above-ground plant parts against biotic stress (Bastias et al., 2017; Constantin et al., 2019; De Palma et al., 2019). Further, they limit their own colonization space by not “overstepping” and “overpowering” the plant in the critical colonization phase.

As the colonization phase continues, the endosymbiotic relationship that confers benefits to both organisms is developed. Host plants maintain endophytic communities in their specific tissues by supplying them with nutrients (Hao et al., 2017). Early in colonization, hosts will assist endophytes in colonization by promoting the expression of genes related to motility, chemotaxis, and adhesion by secreting more attachment proteins, cell-wall re-modeling proteins, and ABC transporters (Coutinho et al., 2015; Pankievicz et al., 2016; Sarkar et al., 2017). For instance, *Azoarcus* sp. BH72 released the type V autotransporters Azo1684 and Azo1653 for its establishment in rice hosts (Sarkar et al., 2017). Then, in turn, the hosts provided tissue sites suitable for endophytic growth (Utturkar et al., 2016; Schmid et al., 2017). This phenomenon indicates that a co-cultured endophyte displays more upregulated genes capable of promoting growth fitness of both organisms than non-co-cultured endophytes (Ambrose and Belanger, 2012; Yi et al., 2017; Zhou et al., 2017; Trovero et al., 2018).

#### Colonization With Promotion of Plant Growth and Metabolite Production

Endophytic microorganisms can promote growth of a plant in addition to accelerating its production of metabolites and enhancing its tolerance to stress. Numerous endophytes promote plant growth, even under stress conditions for hosts, because of this complicated interaction. Vahabi et al. (2015b) reported that the plant gene encoding NRT2.5, which belongs to the nitrate transporter family, was upregulated by approximately fourfold in the root-to-shoot signal transduction process after co-cultivation of *Arabidopsis* with *S. indica*, and this stimulated the growth of *Arabidopsis*. A DSE strain, S16, promoted the growth of sweet cherry by regulating nitrate uptake and plant hormone biosynthesis pathways (Wu et al., 2021). Another DSE strain, T010, also positively influenced plant genes involved in phytohormone and flavonoid metabolism to enhance host plant growth (Wu et al., 2020). The presence of *S. indica* also led to better root development by increasing the expression of indole-3-acetic acid (IAA) biosynthesis genes *AXR1* and *AUX1* in barley (Ghaffari et al., 2016), and the upregulation of genes encoding enzymes related to fatty acid biosynthesis, which resulted in better reproductive growth and improved oil quality of *Brassica napus* (Su et al., 2017). Inoculation of banana (*Musa acuminata*) with the plant growth promoting (PGP) rhizobacterium *Bacillus amyloliquefaciens* Bs006 led to the induction of many genes involved in nutrient absorption and upregulation of the plant cytochrome C (CYTC) gene in common with *Pseudomonas fluorescens* Ps006 which improves for root development (Gamez et al., 2019). Endophytic fungi isolated from *Noccaea* species enhanced the expression of ion transporters and fatty acid biosynthesis related genes to promote plant growth (Ważny et al., 2021).

Apart from the upregulated genes related to plant growth, an endophyte may also influence plants’ primary and secondary metabolite accumulation to increase plant growth. It is known that medicinal plants contain abundant metabolites and endophytes. Some medicinal plants, such as *Salvia miltiorrhiza*, ginseng, and *Echinacea purpurea*, have been studied as model plants for their interactions with endophytes (Maggini et al., 2020; Chu and Bae, 2021; Lu, 2021). In research on *S. miltiorrhiza* Bunge, the endophytic fungi U104 was able to induce the accumulation of tanshinone in the host after its infection (Jiang Y. et al., 2019). Although the endophytic interaction with medicinal plants is rarely studied at the transcriptomic level, some studies have linked these two phenomena. For example, Li et al. (2017a,b) reported that the increment of two metabolite types was associated with thickening of the stem, in comparison with non-inoculated plants. The polysaccharide level in *Dendrobium nobile* rose when *D. nobile* was co-cultivated with MF23 (*Mycena* sp.), as genes involved in photosynthesis, sucrose, fructose, and mannose metabolism were induced, whereas those involved in glycolysis and citrate cycle (TCA) were repressed (Li et al., 2017b). Also, MF23 might stimulate dendrobine biosynthesis by regulating the expression of key genes involved in the mevalonate (MVA) pathway, such as *AACT*, *MVD*, and *PMK*. Moreover, it was found that *Ceratobasidium* sp. AR2 was able to hasten the accumulation of flavonoids in *Anoectochilus roxburghii* by upregulating flavonoid biosynthesis genes (Zhang et al., 2020). In *Atractylodes lancea*, inoculation with *Gilmaniella* sp. AL12 upregulated sesquiterpenoids biosynthesis to improve that plant’s growth (Yuan et al., 2019).

A comparison of microbial effects confirms the growth benefits to hosts from endophytes. Manganiello et al. (2018) used the endophytic fungus *Trichoderma harzianum* and pathogenic fungus *Rhizoctonia solani* to invade tomato, finding that levels of steroidal glycoalkaloids were increased in the *T. harzianum*-colonized tomato, relative to the *R. solani*-colonized host after ingress into the host, which favored plant growth and defense. Besides growth stimulation, the accumulation of bioactive compounds in response to colonization by endophytes also assisted their host in responding to environmental stresses (Herburger et al., 2019; Yan et al., 2019).

#### Colonization With Enhancement of Abiotic Stress

Stress tolerance can also be enhanced by the modulation of phytohormones and reactive oxygen species (ROS) as induced by co-cultivation of plants with endophytes. Under non-stressed conditions, the inoculated endophyte positively regulates stress response genes of the host. The *Trichoderma asperellum*-treated rice plants showed increased expression levels of genes involved in photosynthesis, cell wall, and ROS modulation systems (Doni et al., 2019). Another study showed that, upon infection by *H. seropedicae*, rice activated iron uptake for the promotion of iron homeostasis, and also stimulated the expression of bacterial genes involved in nitrogen fixation, cell motility, and cell wall synthesis; conversely, certain plant defense genes were repressed, which allowed the endophyte to enter and rapidly colonize the intercellular space and xylem (Brusamarello-Santos et al., 2019). Moreover, in barley, endophytic *Serendipita vermifera* led to an extension of the plant protection barrier *via* the specific induction of genes involved in detoxification and redox homeostasis (Sarkar et al., 2019). Similarly, *Epichloë coenophiala* affected the expression of various WRKY transcription factors associated with the enhanced resistance in its host *Lolium arundinaceum* (Dinkins et al., 2017). Taken together, these studies suggest that the symbiosis continues to foster stress resistance in the host.

Stress phytohormones, including their biosynthesis pathways and signal transduction, are major endogenous effectors that modulate plant physiological responses, leading to environmental adaptation. The ROS, which are generated under a variety of abiotic stresses, accumulate as defense activators and act as signaling molecules in plant defense responses at low levels. Under salinity stress, endophytic microorganisms mainly induce genes involved in biosynthesis and signaling of phytohormones (auxin, JA and ET), iron transport, and ROS scavenging (Brotman et al., 2013; Ghaffari et al., 2016; Liu et al., 2017; Qin S. et al., 2018; Eida et al., 2019). Slight differences in various endophyte–plant interactions have also been identified. Colonization by *Glutamicibacter halophytocola* increased the biosynthesis of lignin and flavonoid in leaves (Qin S. et al., 2018). Furthermore, colonization by *B. amyloliquefaciens* was found to upregulate the 1-aminocyclopropane-1-carboxylate (ACC) synthase and ACC oxidase genes in conjunction with the abscisic acid (ABA)-independent pathway of salt tolerance in *A. thaliana* (Liu et al., 2017). Under drought stress, endophytic fungi function differently from endophytic bacteria. For example, *S. indica* enhanced auxin, ABA, SA, and cytokinin levels to enhance the expression of genes involved in the drought stress response of maize hosts (Zhang et al., 2018), while *T. harzianum* improved drought tolerance in rice by modulating the activity of genes for aquaporin and dehydrin, dehydration responsive element binding protein, and superoxide dismutase (SOD) (Pandey et al., 2016). However, in sugarcane plants treated with endophytic bacteria, *Gluconacetobacter diazotrophicus* has suppressed auxin, ABA, and ethylene (ET) hormone pathways in the roots, and activated only the genes involved in ABA-dependent signaling in the shoots (Vargas et al., 2014). In *A. thaliana* colonized by *Pseudomonas chlororaphis*, plant defense-related genes were activated and genes involved in drought response signaling (ABA and ET) were downregulated (Cho et al., 2013). In response to inoculation with *Burkholderia phytofirmans*, potato plants increased their expression of genes involved in extracytoplasmatic function group IV sigma factors, oxidative phosphorylation, transcription regulation, cellular homeostasis, and cell redox homeostasis (Sheibani-Tezerji et al., 2015). Moreover, in a study of the interaction between *E. coenophiala* and tall fescue, the endophyte had a small but potentially important effect on its host transcription that may enhance the plant resistance to stress, which entailed the repression of some genes involved in defense against fungi coupled with the priming of genes that may strengthen drought tolerance in various fungal strain/plant genotype combinations under stress conditions (Dinkins et al., 2019). Yet, surprisingly, little is known about the effects on other abiotic stresses, including cold, heavy metal, and heat stress. *Epichloe gansuensis* increased the biosynthesis of alkaloids and unsaturated fatty acids during the seed germination of *Achnatherum inebrians*, thereby increasing tolerance to cold stress (Chen et al., 2016), while *B. phytofirmans* induced the upregulation of some cold stress-related genes by *Vitis vinifera* (Theocharis et al., 2012). The endophytic smut fungus *Thecaphora thlaspeos*, a biotrophic pathogen with balanced virulence, has the ability to overwinter with its perennial hosts while seemingly suppressing cold acclimation (Courville et al., 2019). In plants colonized by *Curvularia protuberata* that is infected with Curvularia thermal tolerance virus, imposition of heat stress increased the expression levels of genes encoding trehalose phosphatase, betaine aldehyde dehydrogenase, taurine catabolism dioxygenase, and scytalone dehydratase (Morsy et al., 2010), while an endophytic *Thermomyces* strain induced the expression of phenylalanine ammonia-lyase in cucumber under heat stress (Ali et al., 2018). Under heavy metal stress, endophyte–plant interactions can affect several pathways, including metal binding and transporting processes; organic acid metabolic processes; and transporting, transcription factor, sulfate assimilation, DNA repair, and cell-wall metabolic processes (Zhao et al., 2015). The ROS scavengers, including glutathione S-transferases, peroxidase, SOD, and catalase, work to detoxify ROS and to balance the redox homeostasis of the hosts under stress conditions. In addition, levels of heat shock proteins, pyrroline-5-carboxylate synthase and its product, proline, as well as trehalose-6-phosphatase phosphatase and its product, trehalose, were enhanced in symbiosis when compared with non-inoculated plants under the above-mentioned stress conditions (Morsy et al., 2010; Dinkins et al., 2019).

These interconnected modules, pathways, compounds, and system-level patterns of molecular plant–endophyte interactions, from recognition to colonization, have been dissected partially by transcriptomics. However, the sophisticated underlying mechanisms involved in these symbioses make it challenging to distinguish the direct contribution of genetics from indirect factors, including determinants that enable plants to differentiate between endophytes and pathogens and to process information, to stimulate interactome networks, and to engage in signal transduction (Rodriguez et al., 2019). Meanwhile, transcriptomic studies are still constrained by experimental design, transcriptomic technology, costly analysis, and data storage. Furthermore, in some cases, gene annotation is a formidable endeavor due to the lack of high-quality reference genomes, especially those of co-expressed genes from hosts and endophytes, which may be difficult to identify *in planta* due to, e.g., the low abundance of bacterial mRNA (Levy et al., 2018). The study of molecular mechanisms of plant–endophyte interactions is nascent and further investigation is needed to deepen our understanding.

### Metabolomics Applied to the Plant–Endophyte Interaction

Modulation of elemental stoichiometry and the metabolome are promising mechanisms to study plant–endophyte interactions (Rivas-Ubach et al., 2012). Quantitative metabolomics studies permit the analysis of root exudates and microbial signal molecules with high precision (Oldroyd and Downie, 2008; Inceoğlu et al., 2011; Peiffer et al., 2013), thus, clarifying how diverse biomolecules participate in a given cascade and the ensuing response of different microbes. Metabolomic research on plant–endophyte interactions indicates that endophytes, directly or indirectly, can synthesize plant growth regulators, plant secondary metabolites, and defense compounds, all of which are closely connected.

#### Growth-Promoting Metabolites

Endophytic microbes can secrete their own components to facilitate various plant growth regulators, such as IAA, cytokinins, and gibberellins, and also siderophores (Swarnalakshmi et al., 2019). Generally, endophytes may produce phytohormones or phytostimulants and signaling compounds to enhance the growth of their host plants (de Santi Ferrara et al., 2012; Khan et al., 2012; Mehmood et al., 2019; Table 2). *Trichoderma* can produce auxins (Angel Contreras-Cornejo et al., 2009) and secrete auxin-like substances, the plant growth regulator harzianolide, and compounds of unknown function like 6-pentyl-a-pyrone (Vinale et al., 2008). The *B. amyloliquefaciens* RWL-1, a PGP endophyte, can produces high levels of IAA by utilizing methanol (Shahzad et al., 2019). In a recent study of *Bacillus* sp. JC03 secreta, GC-MS identified volatile organic compounds, some of which, 2-ethyl-1-hexanol, tetrahydrofuran-3-ol, and 2-heptanone, affected phytohormone biosynthesis and metabolism to promote plant growth (Jiang C. H. et al., 2019); extracts of *Paecilomyces variotii* had the same effect but also promoted plant defense (Lu et al., 2019). In marine algae, co-cultivation with *S. indica* led to a significant modulation of the amounts of metabolites belonging to the GABA shunt pathway, to ameliorate biomass and lipid production (Bhatnagar et al., 2019). When tall fescue was co-cultivated with different *E. coenophiala* strains, root exudate composition was affected regarding lipids, carbohydrates, and carboxylic acids, and plant growth was increased (Guo et al., 2015). Numerous studies have shown that endophytes can produce siderophores which are then exploited by many plants, such as cowpea, silver birch, and black alder (Maksimova et al., 2018; Albelda-Berenguer et al., 2019). For example, *P. fluorescens* C7 improved iron supply to *A. thaliana* by producing siderophores, thereby promoting plant growth (Vansuyt et al., 2007).

#### Host-Related Metabolites

The chemical diversity of plants is enormous. Plants’ evolution has enabled the biosynthesis of a cornucopia of novel chemicals to enable them to survive and communicate in a complex ecological environment (Wurtzel and Kutchan, 2016). However, metabolome studies tend to focus mainly on small molecules found in a biological sample. Secondary metabolites are small organic compounds that are not only directly involved in normal growth but are also implicated in important plant functions (Table 2). Various endophytes produce different secondary metabolites and mediate an elicitor-induced increase in secondary metabolite biosynthesis in specific species and organs, during different stages of plant development (Zhai et al., 2017). Early work by Stierle et al. (1993) reported that *Taxomyces andreanae* isolated from the bark of *Taxus brevifolia* produced taxol during axenic conditions *in vitro*, thus, drawing attention to the compounds produced by endophytes (Jia et al., 2016). Fungal inoculations were shown to promote the content of reducing sugars, total flavonoids, total phenols, *trans*-resveratrol, and activities of phenylalanine ammonia-lyase in both leaves and berries of grapevine, such that inoculation with the endophytic fungal strains CXB-11 (*Nigrospora* sp.) and CXC-13 (*Fusarium* sp.) affected the metabolome of grapes much stronger than other fungal strains (Yang et al., 2016; Huang et al., 2018). Two endophytes originally isolated from grapevine roots, *B. amyloliquefaciens* SB-9 and *P. fluorescens* RG11, increased the melatonin synthesis as well as abundance of the intermediates 5-hydroxytryptophan, serotonin, and N-acetylserotonin (Jiao et al., 2016). This co-metabolism between plant and endophyte has significant implications in studies of biochemical pathways, particularly those involved in the metabolism of polycyclic and polyaromatic compounds (Horvath, 1972; Chauhan et al., 2008). In a study of the full metabolic potential the endophytic fungus *Botryosphaeria mamane* by addition of histone deacetylase inhibitors, a broad spectrum of metabolites could be identified (Triastuti et al., 2019). An investigation of the response of different members of the plant genus *Hypericum* to 18 biotic elicitors revealed that elicitors of *Fusarium oxysporum* and *Trichoderma crassum* predominantly stimulated the synthesis of naphthodianthrones and emodin, while those from *S. indica* promoted phloroglucinol production (Bálintová et al., 2019). Furthermore, compared to co-cultivation with *S. indica*, its cell wall extract acted as a more potent elicitor for asiaticoside production in *Centella asiatica* (Jisha et al., 2018). Taken together, these studies suggest that microbial endophytes modulate the content and type of secondary metabolite production in their host plants (Lucini et al., 2019).

#### Plant Stress Resistance-Related Metabolites

The scope of metabolomics involves the characterization of all metabolites of an organism under the influence of local environmental conditions. The metabolome varies greatly with alterations in the surrounding environment, which induce direct physiological changes correlated with diverse pathways in an organism (Bundy et al., 2005; Table 2). A study of time-series experiments with *A. thaliana* suggested that metabolic activities respond more quickly than that of transcriptional activities to abiotic alterations in real time (Caldana et al., 2011), demonstrating that metabolome studies are of vital importance. Under saline conditions, IAA concentrations of 0.22–25.58 μg mL^–1^, which can be produced by *Pseudomonas* sp., *Rhizobium* sp., *Enterobacter* sp., *Pantoea* sp., *Marinobacterium* sp., *Acinetobacter* sp., and *Sinorhizobium* sp., were shown to influence germination and seedling growth in wheat (Sorty et al., 2016). In another study of wheat under water deficient conditions, two endophytes, *Acremonium sclerotigenum* and *Sarocladium implicatum*, reduced the levels of stress damage markers and lessened the accumulation of metabolites involved in stress adaptation, particularly *via* changes to 2-oxocarboxylic acid metabolism and ABA levels (Llorens et al., 2019). The *S. indica* improved the adaptation of barley to drought stress by regulating amino acid and soluble sugar metabolism (Ghaffari et al., 2019). Some endophytic bacteria can solubilize phosphorus and/or potassium; for example, strains of *Bacillus* sp., which have phosphate-solubilizing potential, successfully improved the yield and quality of fennel in semiarid saline soil (Mishra et al., 2016). In other studies, Nagabhyru et al. (2013) used ion-exchange chromatography and LC-MS to analyze three colonized and non-colonized clones of tall fescue; colonization affected metabolite levels in that higher levels of free glucose, fructose, trehalose, sugar alcohols, proline, and glutamic acid were found in both shoots and roots after 2–3 days of water deprivation. In addition, asexual *Epichloë* endophytes with the *perA* gene for biosynthesis of the insect feeding deterrent peramine, conferred plant resistance to herbivory (Hettiarachchige et al., 2019).

Endophytes provide a rich source of novel bioactive natural products and their interaction with plants under specific conditions can induce the accumulation of numerous metabolites, which can facilitate the growth of plants and stress resistance. To identify those alterations of metabolite profiles induced either within a system or by a specific gene, metabolomic technology offers a powerful tool to quantitatively and qualitatively detect small molecules and their changes at the plant–endophyte interface, thereby enabling identification of novel metabolites for further endophyte applications.

Nevertheless, there are several challenges associated with conducting a metabolome analysis in plant–microbe systems. The equipment for metabolomic research is less accessible than those of DNA sequencing services. Furthermore, the availability of public metabolite reference databases is still limited, which further complicates the elucidaton and interpretation of metabolite networks and the assignment of detected metabolites to specific organisms (Levy et al., 2018). Furthermore, in the absence of a model compound, the identification of some isomeric or unknown compounds in the metabolite profile remains a challenging task.

## Conclusion

Plant–microbe interactions involving pathogens and symbionts have always been complicated and difficult to understand, because these two categories of microbes share some similarities despite their other differences, for which only multi-omics studies can provide comprehensive global information. Research has addressed the plant–endophyte interactions from the perspective of recognition to signal exchange to stress the tolerance at both their transcriptomic and metabolomic levels. Such studies, based on model plants, have strengthened our understanding of the basic mechanisms underlying the relationship between plants and endophytes, including gene cascades and metabolic pathways, and the accumulation and enhancement of various metabolites, proteins, enzymes, and differentially expressed genes (DEGs). In the recognition phase especially, endophytes will secrete some elicitors/effectors, which are perceived as symbiotic signal molecules by host receptors, initiating the signaling cascades that lead to evading a plant immunity and inducing ISR to enhance coordination *via* phytohormone homeostasis. These secretions of endophytes and receptors of plants could be utilized to enhance plant resistance and moderate fertilizer use towards improving the nutrient captured by plants for enhancing sustainable crop production, which contributes to future applications of plant–microbe interactions for accelerating crops’ improvement in agriculture.

In fact, however, we still lack accurate and complete information on many gene functions and metabolites which could lead to overlooking the potential key elements involved in endophytic recognition and colonization. Little is known about core recognition sites in diverse mechanisms that have arisen in non-model plant–endophyte interactions. Nonetheless, combing multi-omics technologies to study endophyte-treated plants, especially those under various abiotic stress factors, will bridge gaps in our current understanding of the potential physiological development of endophytes. Persistent cultivation-independent problems need to be solved to make better use of endophyte resources (Meena et al., 2017) and cultivation-dependent endophytes. It is expected that a more high-throughput biological data will help to focus efforts upon the study of core recognition sites that will generate huge databases and methodologies for the discovery of myriad unknown genes, microbial species, and metabolites, which will collectively enable us to manipulate endophytes to harness their beneficial effects. High-resolution technology, such as single cell RNA sequencing, spatial transcriptome, and metabolome, will increase in usefulness in the near future. Forward and reverse genetic approaches should be applied to characterize detailed gene functions (Song et al., 2021). Meanwhile, the integration of multi-omics, cultivation, and ecological characterization (metabolic modeling) (Vilanova and Porcar, 2016) will create a new nexus capable of generating “holo-mics.” This is essential for building robust models that reveal both genetic and metabolic potential, as well as the ecology and evolution, of endophytes, the network of the complex interactions of endophytes with their host plants, and other associated microbes, and to let us better understand their respective roles in nature. We anticipate that full applications of these models would contribute to maintaining plant health in a stable living environment in the years to come.

## Author Contributions

JS, LW, and SC reviewed and finalized the manuscript. XC completed the manuscript writing. XC and MS integrated the information of tables, analyzed the data, and made the pictures. All authors reviewed and approved the manuscript.

## Conflict of Interest

The authors declare that the research was conducted in the absence of any commercial or financial relationships that could be construed as a potential conflict of interest.

## Publisher’s Note

All claims expressed in this article are solely those of the authors and do not necessarily represent those of their affiliated organizations, or those of the publisher, the editors and the reviewers. Any product that may be evaluated in this article, or claim that may be made by its manufacturer, is not guaranteed or endorsed by the publisher.
